# Stress Intensity Factors for Embedded, Surface, and Corner Cracks in Finite-Thickness Plates Subjected to Tensile Loading

**DOI:** 10.3390/ma14112807

**Published:** 2021-05-25

**Authors:** Jesús Toribio, Beatriz González, Juan-Carlos Matos, Óscar Mulas

**Affiliations:** Fracture & Structural Integrity Research Group (FSIRG), University of Salamanca (USAL), E.P.S., Campus Viriato, Avda. Requejo 33, 49022 Zamora, Spain; bgonzalez@usal.es (B.G.); jcmatos@usal.es (J.-C.M.); u151273@usal.es (Ó.M.)

**Keywords:** elliptically cracked finite-thickness plate, embedded, surface and corner cracks, imposed displacement, applied tensile load, finite element method, *J*-integral, stress intensity factor

## Abstract

The aim of this study is to obtain the stress intensity factor (SIF) along the crack front of elliptical cracks located in finite-thickness plates subjected to imposed displacement or applied tensile load, for different crack geometries (relative depths and aspect ratios) and crack configurations (embedded, surface, and corner). The SIF was calculated from the *J*-integral, obtained by the finite element method. The results show how the SIF grows with the increase in the relative crack depth and with the decrease in the aspect ratio, with the corner crack being the most dangerous configuration and the embedded crack the most favorable configuration. By increasing the plate length, the SIF rises when the plate is under imposed displacement and decreases when the plate is subjected to applied tensile load, both cases tending towards the same SIF curve.

## 1. Introduction

The solution calculation of the stress intensity factor (SIF) in cracked finite-thickness plates subjected to tension [1,2,3,4] is of great interest in the field of fracture mechanics. The maximum SIF for embedded cracks appears at the point of the crack front closest to the outer surface of the plates [5], while for superficial cracks, it occurs at the point of maximum depth or at the point of intersection with the outer surface, depending on the crack aspect ratio [6,7]. For corner cracks, a quick loss of crack front constraint near the free surfaces seems to be more evident as the crack becomes deeper [8].

For large aspect ratio surface and corner cracks at a semi-circular notch, SIFs are greater for larger crack lengths and for higher notch radii, with them being nearly constant along the crack front for deep surface cracks and for all corner cracks [9]. For cracks located in holes, SIF increases with the ratio between the stress concentrator radius and the plate thickness. In shallow cracks, most of the crack front is generally in a region influenced by the stress concentration of the notch; while in deep cracks, the front lies further from the notch, with a lower stress gradient [10].

When there are two elliptical coplanar cracks, an interaction appears between them such that the SIF depends on the crack depth and the distance between the cracks [11]. Furthermore, the interaction is greater when the cracks are aligned along the minor semi-axis of the ellipses than when they are aligned along the major semi-axis [12,13]. The interaction of non-coplanar parallel cracks produces the appearance of a mixed mode [14].

For deflected and inclined corner cracks in finite-thickness plates under tensile remote loading, decreasing the plate thickness results in the magnification of mixed mode stress intensity factors along the crack front [15]. For inclined surface cracks (mixed mode), the mode-I SIF decreases as the inclination angle increases when the relative depth and the aspect ratio are constant, mode-II being maximum for an inclination angle of 45° [16].

The aim of this research work is to obtain the dimensionless SIF for elliptical cracks in finite-thickness plates subjected to tensile loading, and to compare the results with those of cracked infinite-thickness plates. The main contribution of this paper is to determine how the crack configuration (embedded, surface, or corner), the crack geometry (relative depth and aspect ratio), the plate length, and the mode of solicitation application (imposed displacement or applied tensile load) influence the SIF value.

## 2. Numerical Modeling

**Figure 1:**
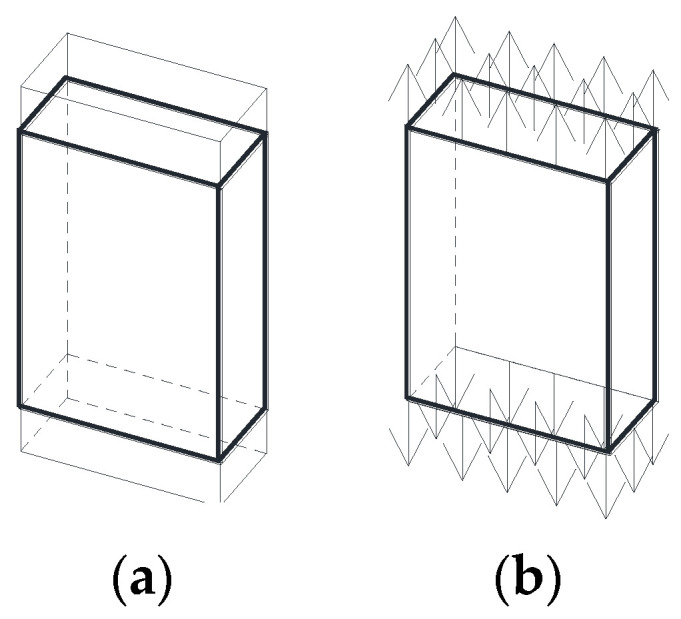
Plate subjected to (**a**) imposed displacement and (**b**) applied tensile load.

**Figure 2:**
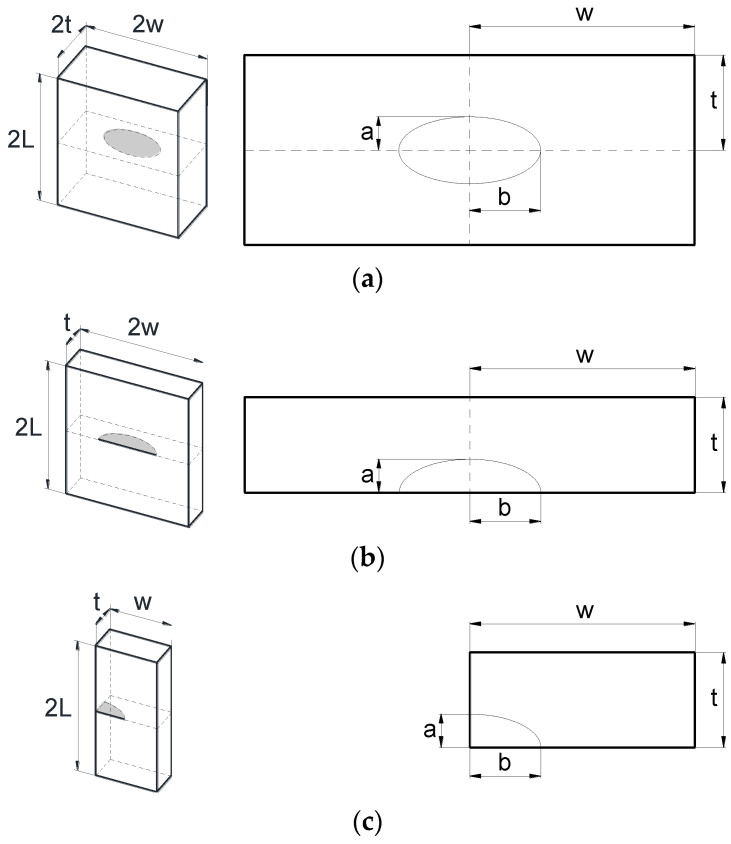
Plate with (**a**) an elliptical embedded crack, (**b**) a semi-elliptical surface crack, and (**c**) a quarter-elliptical corner crack.

The finite element method (FEM) was used, through the MSC Marc commercial software, to obtain the SIF in a cracked finite-thickness plate subjected to imposed displacement (Figure 1a) or applied tensile load (Figure 1b) considering three crack configurations: embedded (Figure 2a), superficial (Figure 2b), and corner (Figure 2c) cracks. The geometry of the crack front was modeled with a pre-defined shape: an ellipse with semi-axes *a* (crack depth) and *b* (crack length).

For the finite-thickness plate, both length (*L*) and width (*w*) are equal, and the thickness (*t*) to width (*w*) ratio was *t*/*w* = 0.1. Several crack geometries, characterized by the relative crack depth (*a*/*t*) and the crack aspect ratio (*a*/*b*), were modeled; specifically, there were twelve crack geometries combining the following values: *a*/*t* = {0.2, 0.4, 0.6, 0.8} and *a*/*b* = {1.0, 0.5, 0.2}. In addition, the results for the finite-thickness plate were compared with those of a large plate with respect to the crack dimensions (infinite-thickness), increasing the plate thickness to *t*/*w* = 1 and using a crack depth value of *a*/*t* = 0.02.

**Figure 3:**
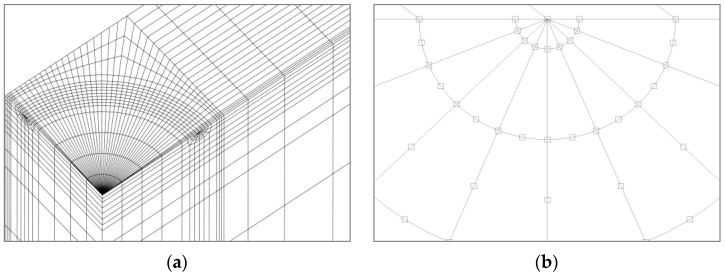
Mesh of the plate: (**a**) cracked surface, and (**b**) detail of the crack tip.

The same meshes with the appropriate boundary conditions according to the symmetry of the problem were used for the modeling of the three configurations studied, which correspond to the eighth part of the plate for the embedded crack, the fourth part of the plate for the superficial crack, and half of the plate for the corner crack (Figure 3a). The mesh was developed with 20-node hexahedron isoparametric elements and full integration, which, at the crack tip, were degenerated elements with the nodes closest to the crack front located at 1/4 (as shown in Figure 3b) to reproduce the stress singularity at these crack front points. The mesh was further refined in the region near the crack front and within it, in the area closest to the *a* and *b* semi-axes, where the edges of some elements measured ~0.025*t*. Boundary conditions were posed according to the solicitation (imposed displacement or applied tensile load) and the specimen symmetries (avoiding the displacement of mesh nodes that coincided with a symmetry plane in its perpendicular direction). A sensitivity analysis was carried out in relation to the mesh size, especially in the area near the crack tip.

The SIF in mode I (*K*_I_) was evaluated through the energy release rate (*G*). In the linear elastic regime and considering that the crack front is under plane deformation conditions (except for the points in contact with the plate outer surface), both parameters are related by the following expression [17]:

(1) $$G=\frac{K_{I}^{2}}{E/\left(1-\nu^{2}\right)}$$

where *E* and *v* are Young’s modulus and Poisson’s ratio, respectively.

For the modeling, the following characteristic properties of the material were used: Young’s modulus *E* = 200 GPa and Poisson’s ratio *ν* = 0.3 (corresponding to steel). The energy release rate (*G*) was calculated through the contour *J*-integral [18].

The dimensionless SIF *Y* (function of dimensionless geometric relations of the cracked plate) is defined with the expression:

(2) $$Y=\frac{K_{I}}{\sigma \sqrt{\pi a}}$$

where the denominator corresponds to the SIF (in mode I) of a through-thickness central crack located in a plate (of dimensions much greater than the crack length 2*a*) subjected to remote tensile stress *σ* [17].

For the case of applied tensile load, the stress *σ* is that placed on the plate ends in the modeling. For the case of imposed displacement, the stress *σ* is calculated from the load *F* at those ends and the section on which it is applied:

(3) $$\sigma =\frac{F}{wt}$$

**Figure 4:**
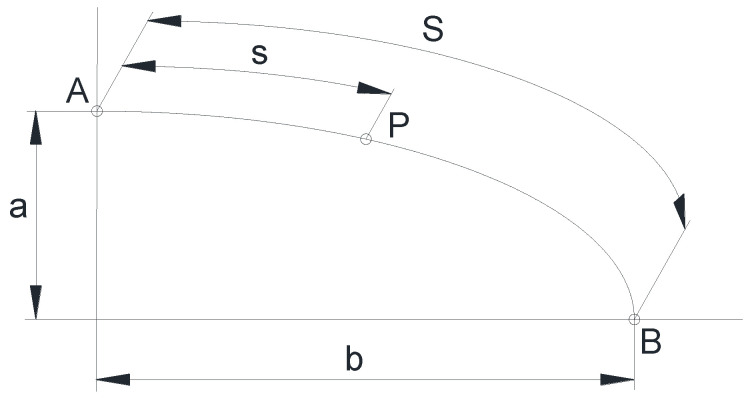
Characterization of a crack front point *P* through the length *s*.

Each point *P* of the crack front was characterized through the dimensionless ratio *s*/*S*, where *S* is the length of a quarter of the ellipse and *s* is the length of the ellipse-arc measured from the point that coincides with the minor semi-axis (crack depth) to the point *P* (Figure 4). The crack front point corresponding to the crack depth (minor semi-axis of the ellipse) has been designated as *A* and the point corresponding to the crack length (ellipse major semi-axis) has been designated as *B*.

## 3. Stress Intensity Factors for the Infinite-Thickness Plate

For an elliptical embedded crack located in an infinite plate subjected to remote tensile loading, the exact solution of the SIF *K*_I_ is given by the Equation [19]:

(4) $$K_{I}=\frac{\sigma \sqrt{\pi a}}{\psi }{(\sin^{2}\phi +\frac{a^{2}}{b^{2}}\cos^{2}\phi )}^{1/4}$$

where the dimensionless SIF depends on the ratio between the semi-axes of the ellipse *a*/*b*, the point of the crack front at which it is calculated (characterized through angle *ϕ*), and the parameter *ψ*.

**Figure 5:**
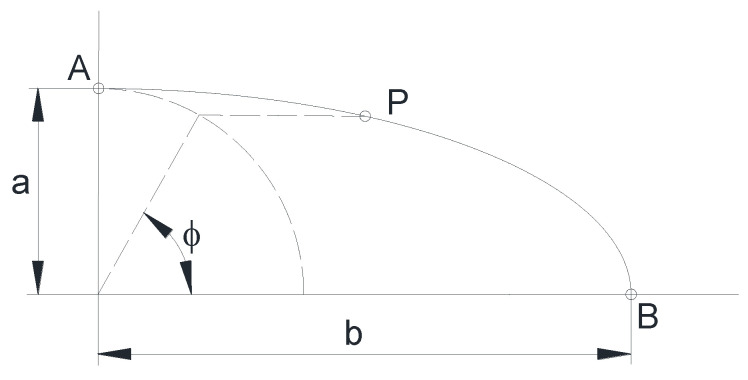
Characterization of a crack front point *P* through the angle *ϕ*.

The elliptical crack front point *P* is determined by the variable *ϕ*, which corresponds to the angle obtained by projecting the point *P* onto a circumference that has the same center as the ellipse and radius *a*, as shown in Figure 5.

The parameter *ψ* is the complete elliptic integral of the second kind and is obtained through the expression [19]:

(5) $$\psi =\int_{0}^{\pi /2}{\left\{1-\left(1-\frac{a^{2}}{b^{2}}\right)\sin^{2}\phi \right\}}^{1/2}d\phi$$

For the particular case in which the crack is circular (semi-axes *a* and *b* of the ellipse have the same value), the equation to obtain the SIF is reduced to the following expression:

(6) $$K_{I}=\frac{2}{\pi }\sigma \sqrt{\pi a}$$

**Figure 6:**
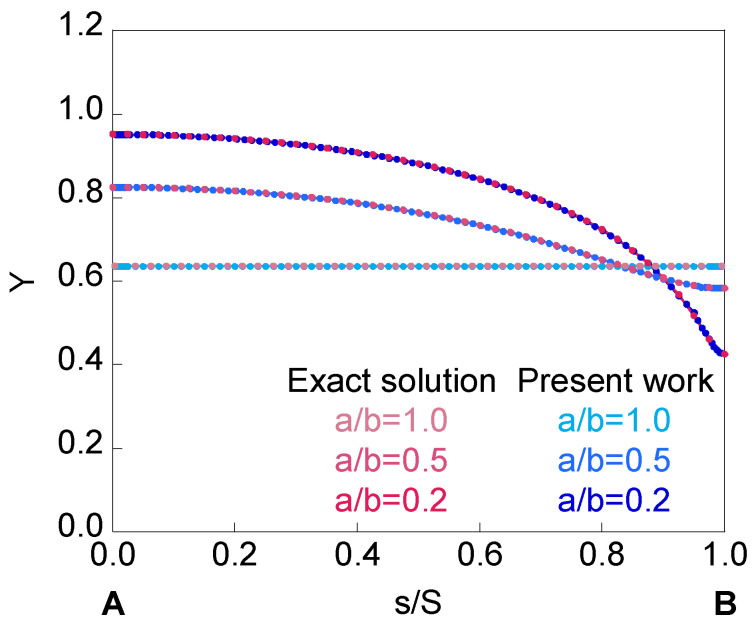
Comparison of dimensionless SIF obtained in the present work with the exact solution for an embedded crack in an infinite-thickness plate.

In Figure 6, the curves *Y*-*s*/*S* obtained in the present work for an embedded crack located in an infinite-thickness plate under applied tensile load are represented together with those corresponding to the exact solution [19]. It is observed to be a very good fit between both results (exact solution and present work) for the aspect ratios *a*/*b* = {1.0, 0.5, 0.2}.

The circular embedded crack shows a constant SIF value along the crack front (it could be considered that the problem presents revolution symmetry). For crack aspect ratios *a*/*b* < 1, SIF increases from point *B* (where the value of the SIF is less than in the circular front case) to point *A* (where the value of the SIF is greater than in the circular front case). The difference between the SIF values for both points *A* and *B* increases as the crack aspect ratio *a*/*b* decreases.

**Figure 7:**
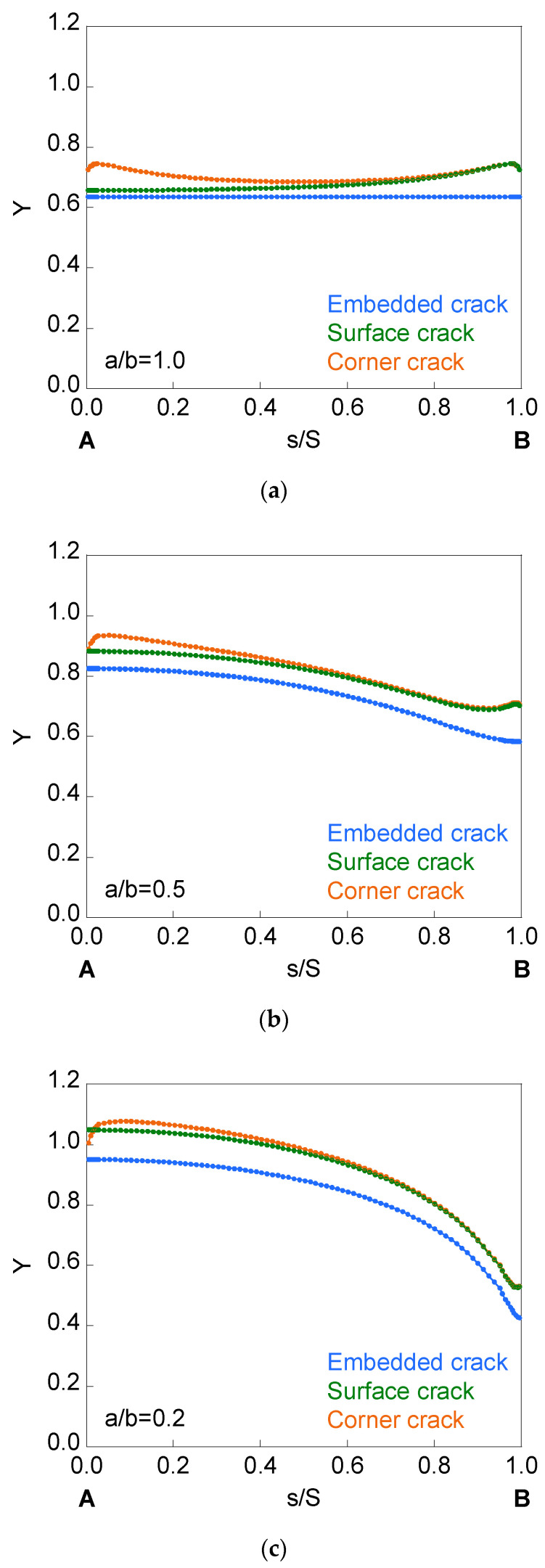
Dimensionless SIF for embedded, surface, and corner cracks in an infinite-thickness plate for (**a**) *a*/*b* = 1.0, (**b**) *a*/*b* = 0.5, and (**c**) *a*/*b* = 0.2.

The curves *Y*-*s*/*S* for an embedded, surface, and corner crack situated in an infinite-thickness plate under imposed displacement or applied tensile load are shown in Figure 7. SIF results are the same for both types of loading (imposed displacement and applied tensile load). The SIF value is higher for the superficial crack than for the embedded crack, the difference between both SIF values being greater at point *B* for the circular crack and at point *A* when the crack becomes straighter (*a*/*b* = 0.2). For the corner crack, the SIF value matches with that of the superficial crack in the part of the crack front near to *B*, increasing its value (with respect to that of the surface crack) in the zone of the crack front near to *A* (with a representation of *Y*-*s*/*S* symmetrical respect *s*/*S* = 0.5 for the circular front crack).

## 4. Stress Intensity Factors for the Finite-Thickness Plate

### 4.1. Crack Geometry Effect

**Figure 8:**
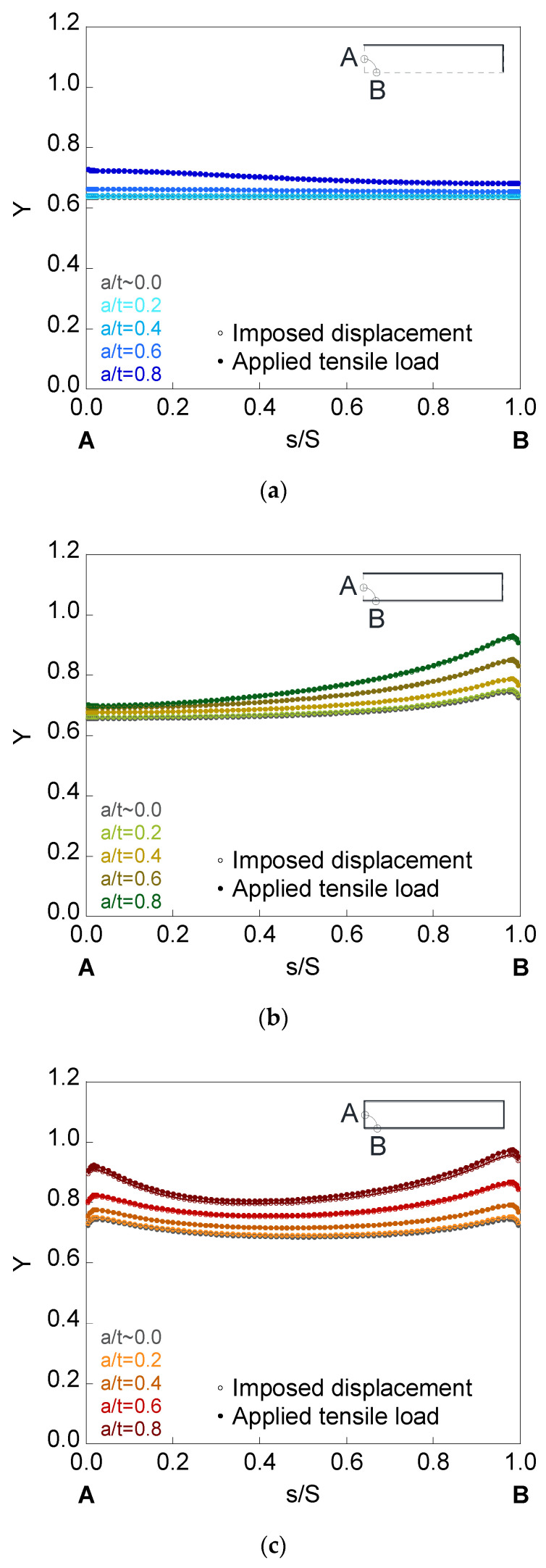
Dimensionless SIF for cracks of relative depths *a*/*t* = {~0.0, 0.2, 0.4, 0.6, 0.8} and aspect ratio *a*/*b* = 1.0 in a finite-thickness plate for the (**a**) embedded configuration, (**b**) superficial configuration, and (**c**) corner configuration.

**Figure 9:**
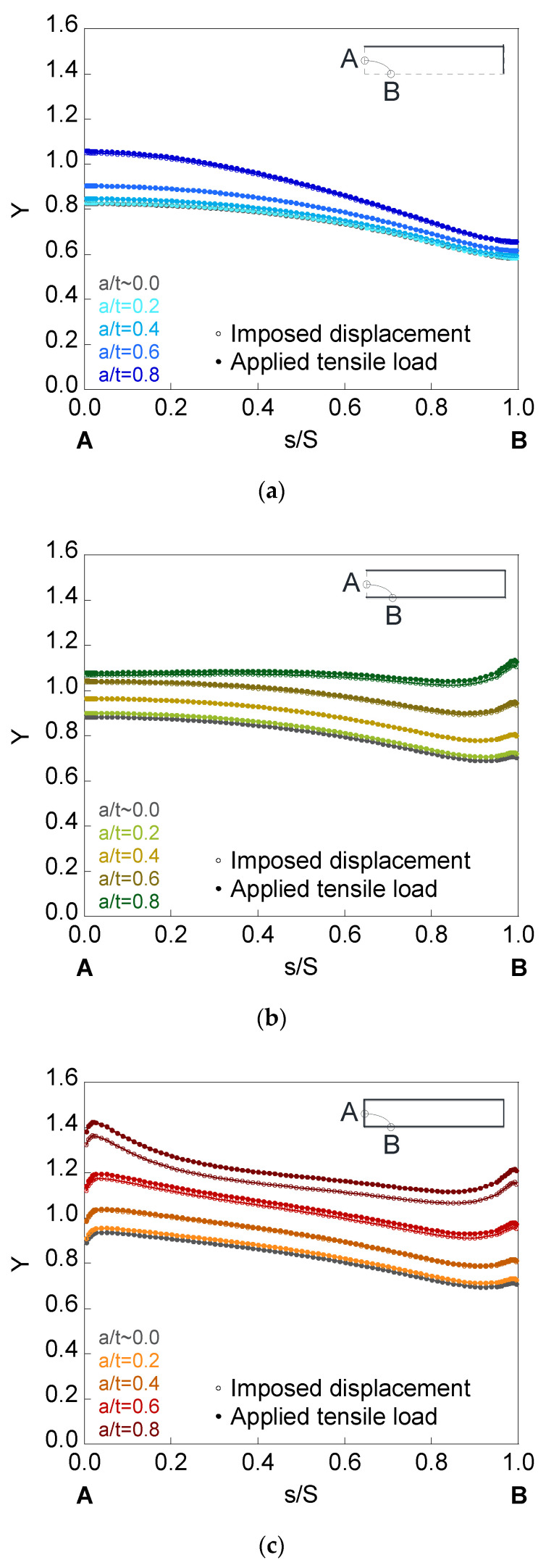
Dimensionless SIF for cracks of relative depths *a*/*t* = {~0.0, 0.2, 0.4, 0.6, 0.8} and aspect ratio *a*/*b* = 0.5 in a finite-thickness plate for the (**a**) embedded configuration, (**b**) superficial configuration, and (**c**) corner configuration.

**Figure 10:**
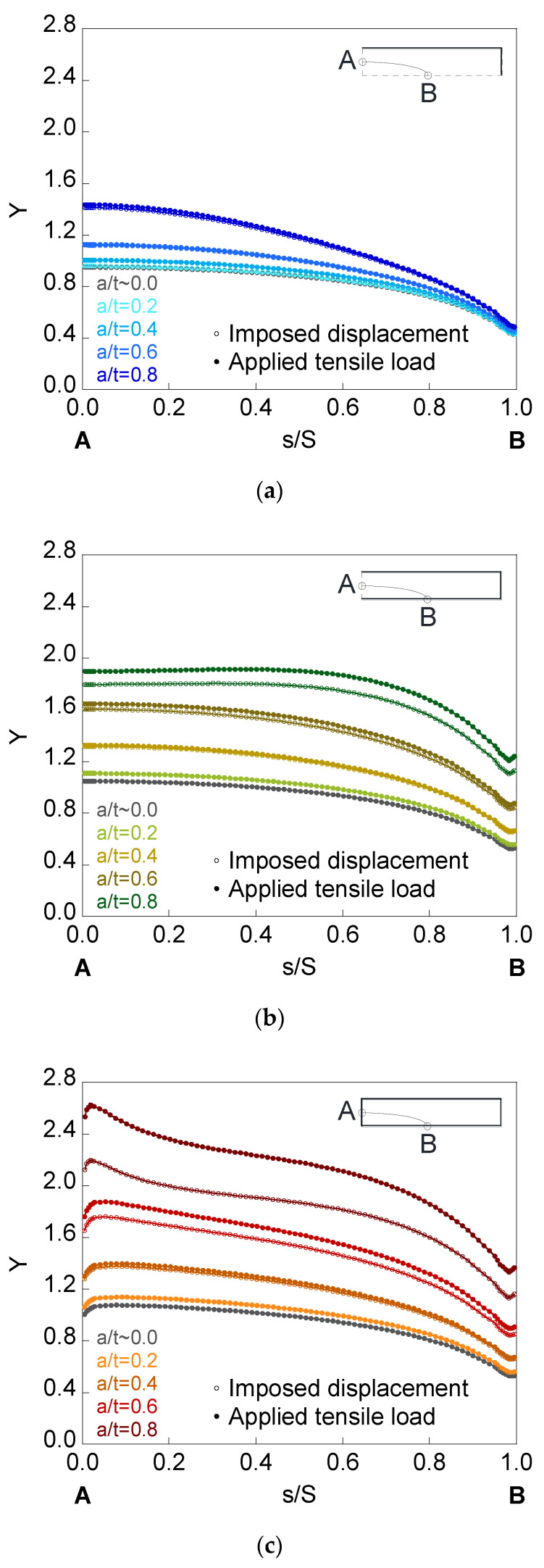
Dimensionless SIF for cracks of relative depths *a*/*t* = {~0.0, 0.2, 0.4, 0.6, 0.8} and aspect ratio *a*/*b* = 0.2 in a finite-thickness plate for the (**a**) embedded configuration, (**b**) superficial configuration, and (**c**) corner configuration.

In Figure 8, Figure 9 and Figure 10, the dimensionless SIF is presented along the crack front (*Y*-*s*/*S* curves) for a finite-thickness plate with an embedded, superficial, or corner crack subjected to imposed displacement or applied tensile load. The elliptical cracks have relative depths of (*a*/*t*) = {~0.0, 0.2, 0.4, 0.6, 0.8} and aspect ratios of (*a*/*b*) = {1.0, 0.5, 0.2}. For all the crack front points, dimensionless SIF values for plates of finite-thickness (colored curves) are greater than for those of infinite-thickness (grayed curves). In addition, its value rises with the increase in the crack relative depth *a*/*t*, with the decrease in the aspect ratio *a*/*b*, and with the existence of the plate outer surface in contact with the crack. Thus, the corner crack is the most dangerous configuration, as indicated by its highest SIF values, while the embedded crack is the most favorable since the SIF values are the smallest.

For embedded cracks, as the crack depth rises, the largest SIF increase occurs in the crack front region next to point *A*. The existence of the plate outer surface in contact with the crack increases the SIF to a greater extent in the area closest to it (except for *a*/*b* = 0.2 and the region close to point *B*). For surface cracks, the largest SIF increase occurs in the area close to point *B*, except for *a*/*b* = 0.2, where the SIF is greater for lower *s*/*S*. For corner cracks, the SIF is greater in the crack front areas close to *B* and *A* than in the intermediate zone (except for *a*/*b* = 0.2, where the SIF increases from *B* to *A*). In addition, for circular corner cracks, the SIF increase is greater in the crack front region next to point *B* than in the crack front region next to point *A*, despite the fact that the latter is closer to the free surface of the plate corresponding to the thickness. This is due to the fact that plate bending is easier along its thickness than along its width.

Regarding the results of the SIF under imposed displacement and under applied tensile load, differences in the SIF between both solicitation modes (SIF is higher under applied tensile load than under imposed displacement) are observed for the deeper cracks with lower aspect ratios. In relation to the crack configuration, the difference in the SIF with the solicitation mode is barely noticeable in the embedded crack, also being more appreciable in the corner configuration than in the surface one.

For surface and corner configurations, the existence of a boundary layer is observed: a small part of the crack front at the intersection with the plate outer surface (point *B* for the surface crack, points *A* and *B* for the corner crack) where the variation of the SIF with the *s*/*S* parameter suddenly changes its trend. This phenomenon may be related to the existence of plane stress at these points of the crack front.

### 4.2. Plate Length Effect

**Figure 11:**
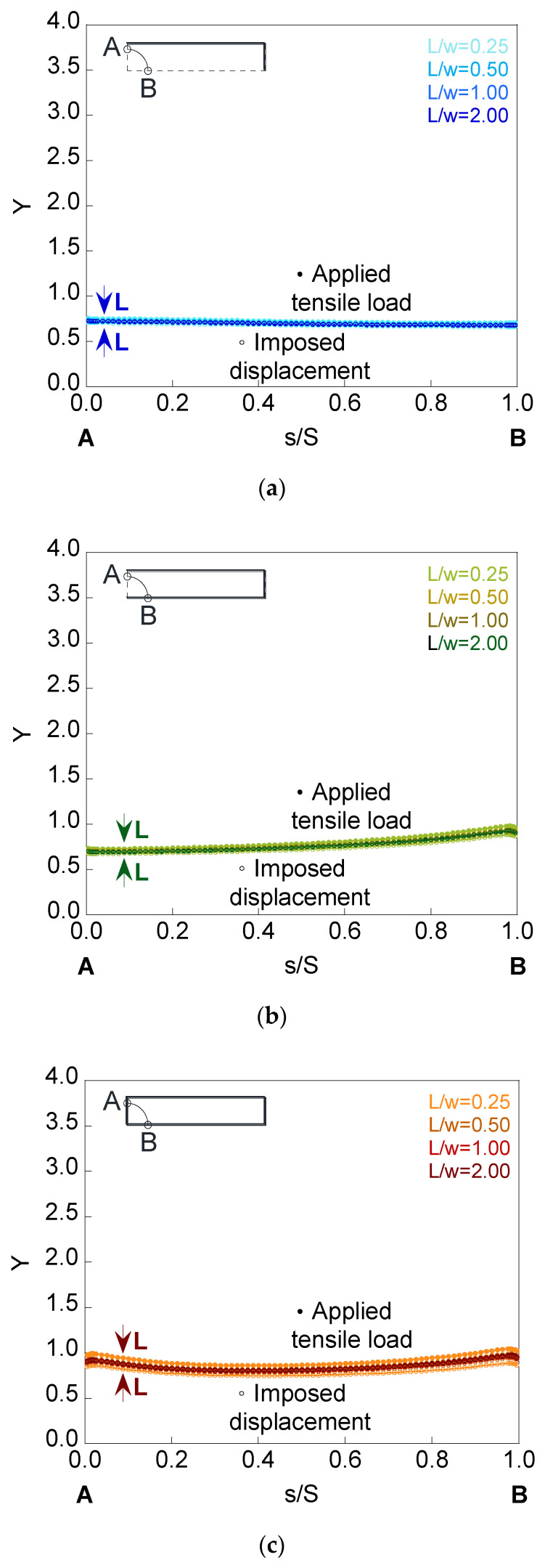
Dimensionless SIF for a crack of relative depth *a*/*t* = 0.8 and aspect ratio *a*/*b* = 1.0 in finite-thickness plates of relative lengths *L*/*w* = {0.25, 0.50, 1.00, 2.00} for the (**a**) embedded configuration, (**b**) superficial configuration, and (**c**) corner configuration.

**Figure 12:**
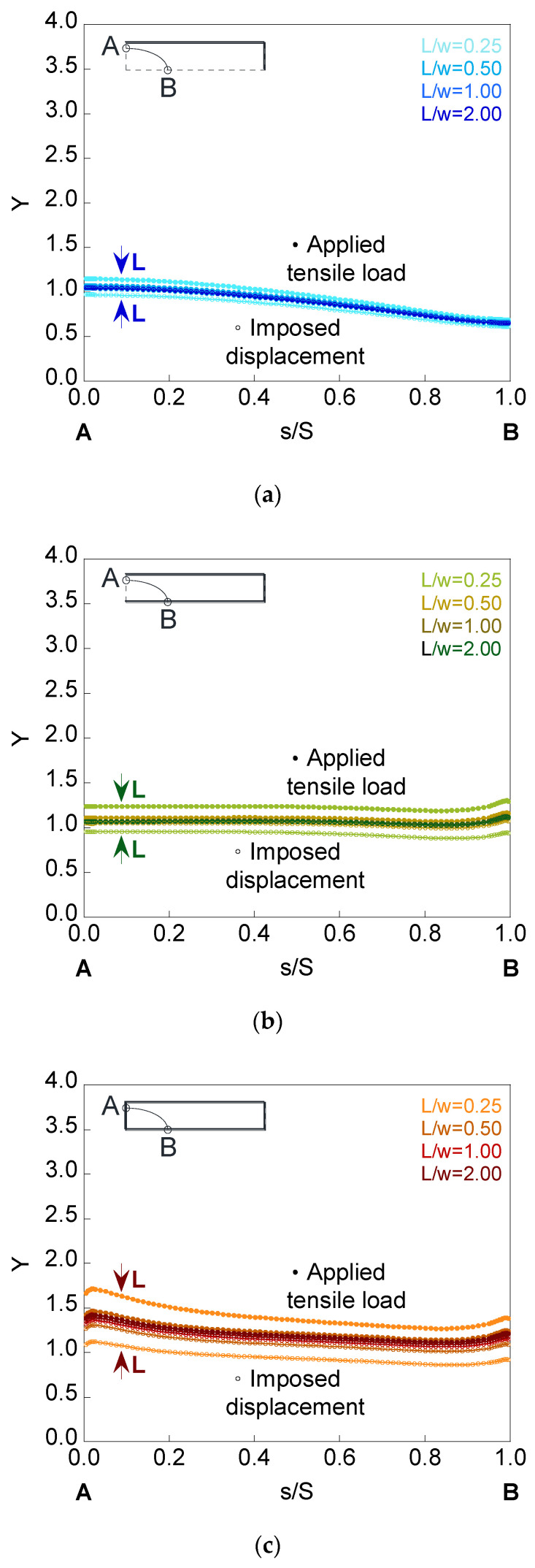
Dimensionless SIF for a crack of relative depth *a*/*t* = 0.8 and aspect ratio *a*/*b* = 0.5 in finite-thickness plates of relative lengths *L*/*w* = {0.25, 0.50, 1.00, 2.00} for the (**a**) embedded configuration, (**b**) superficial configuration, and (**c**) corner configuration.

**Figure 13:**
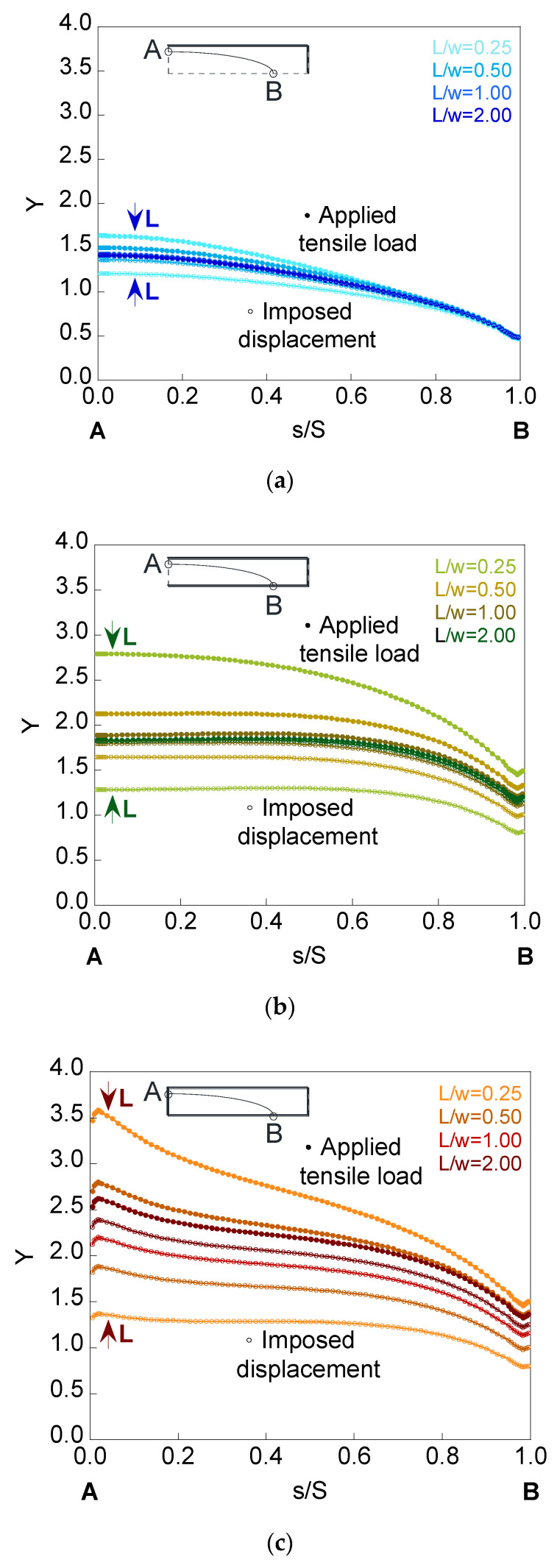
Dimensionless SIF for a crack of relative depth *a*/*t* = 0.8 and aspect ratio *a*/*b* = 0.2 in finite-thickness plates of relative lengths *L*/*w* = {0.25, 0.50, 1.00, 2.00} for the (**a**) embedded configuration, (**b**) superficial configuration, and (**c**) corner configuration.

To analyze the influence of the plate length *L* on the SIF, the deepest cracks with the embedded, superficial, and corner configurations were used. Figure 11, Figure 12 and Figure 13 show the dimensionless SIF along the crack front (*Y*-*s*/*S* curves) for elliptical cracks of relative depth *a*/*t* = 0.8 and aspect ratios *a*/*b* = {1.0, 0.5, 0.2} localized in finite-thickness plates of relative lengths *L*/*w* = {0.25, 0.50, 1.00, 2.00}.

It is observed how, as the plate length increases, the SIF value rises when the plate is under imposed displacement and decreases when the plate is subjected to applied tensile load, the SIF of both cases matching from a certain value of the plate length. The critical length, from which the results do not change with increasing length, depends on the crack configuration, the crack geometry, and the mode in which the load is applied. Thus, decreasing the aspect ratio produces a greater variation in the SIF results for the analyzed plate lengths. In relation to the crack configurations, the greatest difference occurs in the corner configuration and the smallest in the embedded one. The cause may be due to the ability to redistribute stresses and to the bending that could occur in the plate for the surface and corner configurations, being greater in the latter.

## 5. Conclusions

The following conclusions have been obtained in this research work in which the SIF has been calculated in elliptically cracked finite-thickness plates subjected to imposed displacement or applied tensile load:

(i) The existence of finite-thickness (in relation to infinite-thickness) significantly increases the dimensionless SIF when the relative crack depth (*a*/*t*) increases or the crack aspect ratio (*a*/*b*) decreases.
(ii) The corner crack shows the highest SIF values (it being the most dangerous configuration), while the embedded crack shows the smallest SIF values (it being the most favorable configuration).
(iii) The presence of the plate outer surface in contact with the crack increases the SIF to a greater extent in the area closest to it, except for the crack aspect ratio *a*/*b* = 0.2 and the region close to point *B*.
(iv) There are variations of the SIF with plate length for large relative crack depths and small crack aspect ratios, the corner configuration presenting the largest differences and the embedded one exhibiting the smallest ones.
(v) By increasing the plate length, the dimensionless SIF rises when the plate is under imposed displacement and decreases when the plate is subjected to applied tensile load, both cases tending towards the same SIF curve.

## Data Availability

Not applicable.

## List of Symbols

*a*	Crack depth
*a/b*	Crack aspect ratio
*a/t*	Relative crack depth
*A*	Crack front point corresponding to the crack depth
*b *	Crack length
*B*	Crack front point corresponding to the crack length
*E*	Young’s modulus
*F*	Load applied at the plate ends
*ϕ*	Angle characterizing a crack front point
*G*	Energy release rate
*J*	*J*-integral
*K*_I_	Stress intensity factor in mode I
*L*	Plate length
*L/w*	Relative plate depth
*ν*	Poisson’s ratio
*P*	Crack front point
*s*	Ellipse-arc length characterizing a crack front point
*S*	Quarter-ellipse length
*σ*	Load applied at the plate ends divided between the plate cross-section
*t*	Plate thickness
*w*	Plate width
*Y*	Dimensionless stress intensity factor
*ψ*	Complete elliptic integral of the second kind
